# Depression impact on PTSD in Cancer patients through serial mediation of hope and perceived social support

**DOI:** 10.1038/s41598-025-09908-w

**Published:** 2025-07-09

**Authors:** Chen Zhang, Jingjing Wang, Yunhui He, Kaili Wang, Chonghan Wang, Luxiang Zhang, Xiaofei Wu, Shuangquan Liang, Xiaoyan Wu, Yujie Wei, Mingjie Zhang, Wenjuan Wang

**Affiliations:** 1 School of Mental Health, Bengbu Medical University, Bengbu, 233030 Anhui China; 2 Bengbu Medical University, Bengbu, 233030 Anhui China; 3 The First Affiliated Hospital of Bengbu Medical University, Bengbu, 233004 Anhui China

**Keywords:** Cancer, Post-traumatic stress disorder, Depression, Perceived social support, Hope, Cancer, Psychology

## Abstract

Previous studies have only established a strong association between depression and post-traumatic stress disorder in cancer patients, without clarifying the potential mechanisms underlying this relationship. This study examines the relationship between depression and PTSD(Post-Traumatic Stress Disorder) in cancer patients, focusing on the sequential mediation of hope and perceived social support. A cross-sectional study was conducted at oncology wards in an Anhui tertiary hospital between February-August 2024. The investigation employed four instruments: PTSD checklist-civilian version, self-rating depression scale, perceived social support scale, and herth hope index. Analysis included 192 cancer patients, after removing 8 invalid responses from 200 initial participants. Treating depression as the predictor variable in cancer patients revealed a robust association with PTSD symptoms. (*β* = 0.620, *t* = 10.904, *p* < 0 0.001, *R*^2^ = 0.385). A small indirect effect was observed in the association between depression and post-traumatic stress disorder in cancer patients. On one hand, perceived social support demonstrated a mediating effect between depression and PTSD (*SE* = 0.024, *Z* = 2.883, 95% *CI* [0.007, 0.101]). On the other hand, hope alone did not show a significant mediating effect (*SE* = 0.039, Z = -2.211, 95% *CI* [-0.148, 0.006]). However, the serial mediation model confirmed depression’s impact on Post-traumatic stress disorder operated through sequential effects of hope and perceived social support (*SE* = 0.015, *Z* = 2.934, 95% *CI* [0.004, 0.064]).The proportion of the total effect mediated was approximately 3.13%. Depression significantly associates PTSD symptoms in cancer patients through the sequential mediation of hope and perceived social support. These findings suggest that integrated interventions targeting both hope enhancement within social contexts and social support improvement may be more effective than single-component approaches.

**Supplementary Information:**

The online version contains supplementary material available at 10.1038/s41598-025-09908-w.

## Background

 Cancer represents one of the most critical threats to human health globally. Reports from 2022 revealed 20 million new cases and 9.7 million fatalities, while estimates suggest new cases will reach 35 million by 2050^1^. Moreover, the mental health of cancer patients warrants significant attention. Studies have shown that psychiatric disorders are significantly more prevalent among cancer patients, with a higher likelihood of being diagnosed with mental disorders compared to individuals without cancer^2,3^.

Post-traumatic stress disorder (PTSD) is a delayed and long-lasting mental disorder caused by exposure to extraordinarily threatening or catastrophic psychological trauma^4^. Additionally, cancer, as a life-threatening disease, along with its diagnosis, treatment, and associated side effects, can serve as a traumatic stressor and in turn, potentially lead to the development of PTSD in patients^5,6^. Evidence suggests that the incidence of PTSD is higher in cancer patients compared to the general population^7,8^. At the same time, the quality of life of cancer patients is also low and shows a significant negative correlation with their PTSD condition^9^. There are numerous predictors of cancer-related PTSD symptoms, including various psychological and social factors^10^. Emerging evidence indicates that depression, perceived social support, and hope are critical correlates of PTSD among cancer patients^11,12^.

Depression, which accounts for a significant proportion of the various psychiatric and psychological disorders that cancer patients may experience, has a profound impact, with elevated depression scores persisting even 12 to 26 years after diagnosis^13^. The comorbidity of female-related cancers and depression is notably high^14^. Evidence shows that pregnant women with malignant tumors have a 49% higher likelihood of developing depressive or anxiety disorders compared to pregnant women without malignancies^15^. Among breast cancer patients, the prevalence of depressive disorder reaches 32.2%^16^. The relationship between cancer and depression is underpinned by several key pathological mechanisms. Inflammatory factors contribute significantly to cancer patients’ depressive disorders through three mechanisms: kynurenine pathway activation, HPA axis dysfunction, and glutamatergic pathway-induced excitotoxicity^17^. Conversely, depression is a strong predictor of cancer incidence, mortality, and recurrence^18–20^. Cancer patients experiencing major depression show reduced survival outcomes^21^.

Depressive disorder frequently co-occurs with PTSD and has been observed across various types of cancer^22,23^. Compared to those with only one psychiatric disorder, cancer patients with both depressive disorder and PTSD experience more severe negative effects. These patients typically report greater levels of fatigue, sleep disturbances, and cancer-related symptoms, as well as higher levels of pain^24,25^. Research suggests that this pattern of comorbidity is heterogeneous, with core belief challenges and intrusive rumination effectively distinguishing PTSD symptoms from depressive patterns, while social support differentiates the boundary between low and moderate symptom intensity^26^. In cancer patients, the relationship between depressive disorder and PTSD extends beyond mere comorbidity; there is an underlying connection between the two. Their co-occurrence in cancer patients is significantly correlated, with a clear linear relationship observed between their symptom scores^27,28^. Research indicates that depression serves as a robust predictor of PTSD among cancer patients, particularly when coupled with low social support and elevated psychological distress^29^. Research indicates that depressive disorders increase the risk of PTSD development by 46.4% specifically in gastric cancer patients^30^. In a study where cancer patients were stratified into four groups based on depressive syndrome manifestation, findings revealed that cohorts exhibiting more severe depressive symptoms demonstrated correspondingly higher severity in both PTSD and anxiety disorders^31^. Among cancer survivors, depression has been identified to be markedly associated with PTSD subdimensions, such as hypervigilance, suggesting that the persistent effects of depression may exacerbate trauma-related symptoms^32^. Early identification and intervention of depression are crucial for mitigating the risk of PTSD in cancer patients^33^.

Hope is considered to be significantly positively correlated with perceived social support^34^. Among oral cancer patients, hope exhibits inverse associations with PTSD symptoms while mediating perceived stress-PTSD relationships^12^. It is beneficial to cultivate a forward-looking, hopeful mindset to buffer against the impact of trauma. Higher levels of hope are associated with enhanced emotional regulation and coping mechanisms, enabling patients to process their cancer experience with reduced psychological distress^35^. PTSD symptoms exhibit varying correlations with social support across cancer treatment stages. While initially impacting re-experiencing and numbness symptoms, this relationship encompasses all symptoms at six months post-treatment, ultimately focusing solely on numbness after one year^36^. Patients who perceive higher levels of social support demonstrate enhanced stress coping capabilities and are better equipped to reframe their experiences, consequently diminishing the severity of trauma-related symptoms^37^.

Attribution Theory suggests a sequential pathway from depression to PTSD, mediated by hope and perceived social support. Negative attributional patterns among depressed individuals, characterized by internal, stable, and global attributions, may generate pessimistic expectations in cancer patients, diminishing hope and altering social support perceptions. Consequently, reduced psychological resources may increase vulnerability to PTSD symptomatology^38^.

Research examining the comprehensive relationships among these four variables remains insufficient. While previous studies have typically investigated isolated or paired variables, the understanding of their dynamic interactions is incomplete. Furthermore, the direct and indirect pathways from depression to PTSD remain unexplored, particularly regarding the potential mediating effects of hope and perceived social support. Accordingly, this investigation aims to delineate the relationship between depression and PTSD by examining the mediating roles of hope and perceived social support. Specifically, we propose the following hypotheses for cancer patients: (1) Depression is significantly correlated with post-traumatic stress disorder. (2) Hope mediates the relationship between Depression disorder and post-traumatic stress disorder. (3) Perceived social support mediates the relationship between Depression disorder and post-traumatic stress disorder. (4) Hope and perceived social support play a serial mediating role in the relationship between Depression disorder and post-traumatic stress disorder.

## Methods

### Participants

Using a convenience sampling method, the study selected cancer patients who were hospitalized for treatment in cancer-related wards at a Grade A tertiary hospital in Anhui Province from February 2024 to August 2024.

Inclusion criteria: ①Hospitalized cancer patients with pathological or cytological diagnostic evidence, ② age ≥ 18 years, ③ clear consciousness and no communication barriers with investigators, ④ life expectancy of more than 3 months as determined by two physicians, ⑤ consent to participate in this study. Exclusion criterion: Patients with a history of psychiatric disorders.

Variance analysis revealed age-dependent patterns in PTSD manifestation among cancer patients. cancer patients in the young adult group (21–40 years) demonstrated the highest levels of PTSD, while those in the middle-aged group (41–60 years) and early elderly group (61–70 years) exhibited moderate PTSD levels. In contrast, patients in the late elderly group (71–80 years) and oldest old group (81–100 years) showed lower levels of PTSD. Overall, younger age groups were connected with higher levels of PTSD. Notably, age did not exhibit significant differences for the other variables.

T-test analysis showed gender equivalence in depression and hope levels. However, males demonstrated significantly higher social support perception and lower PTSD scores than females. Due to notable variations in key demographic factors, such as age and gender, these variables were included as analytical covariates. In addition, our study only included solid tumors and did not involve hematologic cancers. In these, Lung 22.63%, Stomach 18.42%, Breast 18.95%, Liver 14.21%, Colorectal 8.42%, Other 13.16%.

**Fig. 1:**
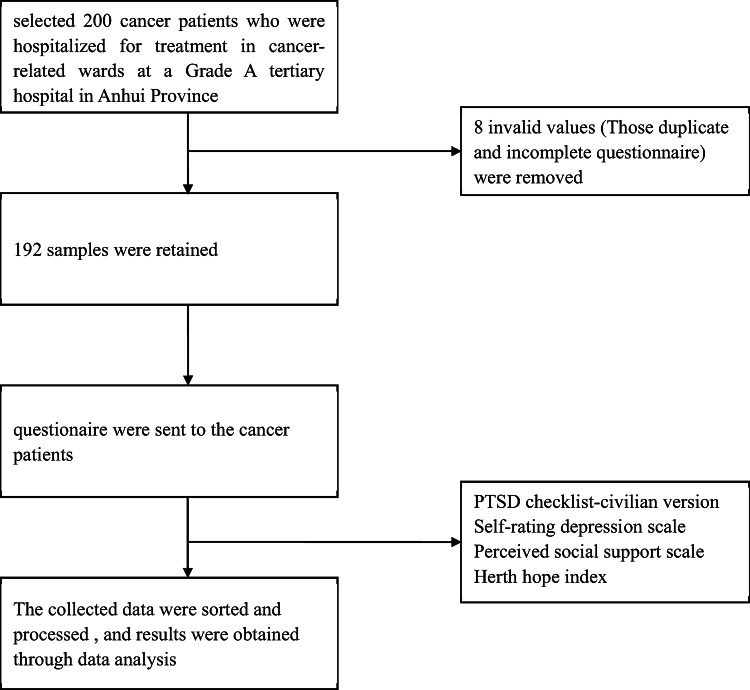
The flowchart of the study.

Participants were fully briefed on study objectives, methods, and confidentiality measures before providing written consent. The research adhered to strict ethical protocols protecting personal data and responses. All methods were performed in accordance with the relevant guidelines and regulations. Figure 1 illustrates the study workflow.

### Measures

Cancer patients were required to provide their demographic information, including age, gender, place of residence, occupation, educational level and Marital Status.

#### PTSD checklist-civilian version

The instrument features 20 questions that assess four components: intrusive experiences, avoidance patterns, cognitive-mood changes, and arousal states. Items are scored on a 0–4 scale (not at all - extremely). The scores ranged from 0 to 80. Multiple validation studies confirm its reliability (Cronbach’s α = 0.902).The PTSD Checklist-Civilian Version (PCL-C) was utilized to assess PTSD symptoms in cancer patients, whose exact cutoff score used in the PCL-C was ≥ 33. This widely validated self-report instrument facilitates efficient screening in oncology settings where comprehensive assessment resources may be limited. While structured clinical interviews such as the CAPS-5 provide more thorough evaluation, they require specialized training, greater time commitment, and additional clinical resources. The PCL-C was selected for its established reliability, efficiency in administration, and capacity to screen larger sample populations while maintaining adequate diagnostic sensitivity, making it particularly suitable for medical settings where physical health concerns typically take precedence.

#### Self-rating depression scale

Originally created by Zung in 1965 and adapted to Chinese context by Shuliang (1999), the SDS measures depression through 20 items. The assessment employs a 4-point scale (1 = never, 2 = sometimes, 3 = means often, 4 = always), with ten items requiring reverse scoring.Total depression scores ranged from 20 to 80, with a cutoff value of 50. Total scores correlate positively with depression levels. The scale showed robust reliability (Cronbach’s α = 0.87).

#### Perceived social support scale

Jiang Qianjin’s modified Social Support Scale measures three domains: friend, family, and other support networks, comprising 12 items. Each item utilizes a 7-point Likert format (1 = “strongly disagree”, 7 = “strongly agree”), yielding total scores between 12 and 84. Higher scores reflect stronger perceived support. The scale demonstrated excellent reliability (α = 0.953).

#### Herth hope index

Hope levels were measured using the Herth Hope Index (HHI).The Chinese version was revised by Zhao Haiping et al. The scale consists of 12 items, divided into three dimensions: positive attitudes (4 items: items 1, 2, 6, 11), positive actions (4 items: items 4, 7, 10, 12), and close relationships (4 items: items 3, 5, 8, 9). Each item uses 4-point ratings (strongly disagree = 1 to strongly agree = 4), with two reverse-coded items. Scores (range: 12–48) indicate hope levels (low:12–23; moderate:24–35; high:36–48). The Chinese version showed excellent reliability (Cronbach’s α = 0.850).

### Procedure

Before the commencement of the study, all participants and their guardians were required to provide written informed consent. Cancer patients completed these psychological assessments in the hospital ward. The questionnaires were distributed manually, and patients responded on paper forms. To optimize high-quality data measurement, each patient received guidance from at least two researchers before they began completing the questionnaires. These researchers addressed participant inquiries and provided guidance throughout survey completion. For cancer patients with literacy impairments, researchers obtained their responses by reading the questionnaire items to them and marking their answers accordingly their answer. During the process, researchers maintained objectivity and refrained from using any suggestive or leading language.

### Data analysis

Data analysis utilized SPSS 20, with the PROCESS macro (Model 6) testing chain mediation through 5,000 bootstrap iterations^39,40^. Heteroscedasticity consistent standard errors were used in the analysis. two-tailed tests, and bias-corrected confidence intervals (CIs) were employed. Chain mediation represents a sequential process in which mediators successively link the independent variable to the dependent variable through multiple intermediary factors. This analytical approach proceeded as follows: (a) Specifying key variables: depression (independent), PTSD (dependent), and mediators (social support, hope); (b) Implementing PROCESS Model 6 to examine the sequential mediation structure; (c) Establishing 5,000 bootstrap samples for indirect effect calculation and confidence interval estimation; and (d) Computing path coefficients to evaluate each mediation step. The mediation was considered significant when the 95% confidence intervals of coefficients did not contain zero^39^. Given the notable variations in demographic variables such as age and gender, these were included as covariates to control for their potential influence.

To evaluate common method bias, Harman’s single-factor test was utilized. The results indicated that four factors had eigenvalues exceeding 1, with the first factor accounting for 28.04% of the variance, well below the 40% threshold. These findings suggested that common method bias did not substantially affect this study. Additionally, the psychological scales had differences in their dimensions. To ensure that variables with different dimensions had equal influence in the model, the key variables were standardized. This prevented bias caused by the discrepancies in dimensions. Standardization also indirectly reduced multicollinearity from interaction terms or polynomial terms. As a result, the model’s numerical stability and interpretability improved and the regression coefficients provided were from raw scores.

## Results

### Differential analysis of gender and age in this study

Tables 1 and 2 demonstrated differential analysis of gender and age in current study. Independent samples t-tests showed considerable gender disparity in trauma and perceived social support scores. Analysis revealed that females exhibited significantly higher trauma scores (*M* = 0.24) compared to males (*M* = 0.15; *t* = 3.114, *p* = 0.002). Conversely, females reported significantly lower perceived social support scores (*M* = 0.60). relative to males (*M* = 0.68; *t* = −2.121, *p* = 0.036). Analysis of variance (ANOVA) revealed significant age-related differences in trauma scores (*F* = 2.755, *p* = 0.029).

**Table 1:** Analysis of variance on age Difference.

	Age(M ± SD)					F	*p*
	Young adults(*n* = 7)	Middle aged(*n* = 78)	Young-old adults(*n* = 62)	Middle-old adults(*n* = 39)	Oldest-old adults(*n* = 6)		
Depression	0.30 ± 0.14	0.36 ± 0.12	0.35 ± 0.13	0.33 ± 0.10	0.32 ± 0.09	0.666	0.616
PTSD	0.25 ± 0.22	0.21 ± 0.19	0.18 ± 0.18	0.11 ± 0.11	0.11 ± 0.05	2.755	0.029*
Hope	0.79 ± 0.40	0.69 ± 0.27	0.70 ± 0.29	0.78 ± 0.38	0.78 ± 0.20	0.833	0.506
Perceived social support	0.61 ± 0.34	0.65 ± 0.23	0.63 ± 0.22	0.68 ± 0.22	0.73 ± 0.18	0.542	0.705

Note.Young adults represents ages 21–40, Middle aged represents ages 41–60, Young-old adults represents ages 61–70, Middle-old adults represents ages 71–80, Oldest-old adults represents ages 81–100 * *p* < 0.05 ** *p* < 0.01.

**Table 2:** Independent samples t-Test for gender Differences.

	Gender(M ± SD)		t	*p*
	Female(*n* = 70)	Male(*n* = 122)		
PTSD	0.24 ± 0.20	0.15 ± 0.16	3.114	0.002**
Depression	0.36 ± 0.13	0.34 ± 0.11	1.491	0.138
Hope	0.72 ± 0.34	0.72 ± 0.28	0.165	0.869
Perceived social support	0.60 ± 0.26	0.68 ± 0.20	−2.121	0.036*

Note. * *p* < 0.05 ** *p* < 0.01.

### An analysis of the correlation among age, gender, place of residence, occupation, educational level

Table 3 presents the correlation coefficients among gender, age, place of residence, occupation, educational level, depression, PTSD, perceived social support and hope.

**Table 3:** Correlation, and reliabilities for all Variables.

	Depression	Hope	PTSD	Perceived social support	Education level	Occupation	Place of residence	Marital Status	Age	Gender
Depression	1									
Hope	−0.427**	1								
PTSD	0.620**	−0.219**	1							
Perceived Social Support	−0.457**	0.519**	−0.399**	1						
Education level	−0.056	0.032	0.026	0.050	1					
Occupation	−0.008	−0.054	0.007	0.100	0.198**	1				
Place of residence	0.050	−0.016	0.040	0.022	0.211**	0.195**	1			
Marital Status	−0.059	0.021	−0.097	0.060	−0.042	0.129	−0.050	1		
Age	−0.062	0.090	−0.228**	0.072	−0.045	−0.218**	−0.160*	0.052	1	
Gender	0.108	0.013	0.235**	−0.162*	−0.104	−0.009	0.016	0.029	−0.095	1

Note. * *p* < 0.05 ** *p* < 0.01.

Regression modeling examined PTSD as the outcome variable, controlling for demographic covariates including gender, age, residence location, occupation, and education level. Depression was designated as the independent variable, and the results demonstrated a important influence of depression on PTSD. (*β* = 0.620, *t* = 10.904, *p* < 0 0.001, *R*^2^ = 0.385), indicating that higher depression is associated with greater PTSD.

### The effect of depression on post-traumatic stress disorder: the chain mediating role of hope and perceived social support

The evaluation of sequential mediating mechanisms revealed how initial factors led to the development of PTSD symptoms. The analysis illustrated that depression exhibited a significant negative effect on hope (*B* = −0.513, *β* = −0.427, *t* = −6.467, *p* < 0.001) and perceived social support (*B* = −0.485, *β* = −0.269, *t* = −4.115, *p* < 0.001). In terms of perceived social support, hope (*B* = 0.611, *β* = 0.407, *t* = 6.228, *p* < 0.001) significantly positively influenced this variable.

**Fig. 2:**
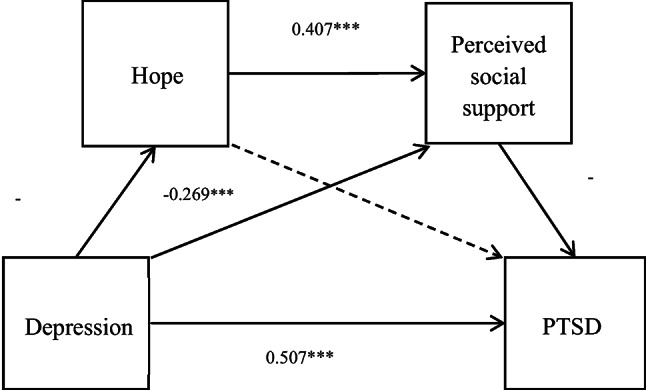
The serial mediation role of hope and perceived social support in the relationship between depression and PTSD. Note. 1. Age → PTSD : *β* = 0.209***; gender → PTSD: *β* = −0.118*. 2.* *p* < 0.05, ** *p* < 0.01, *** *p* < 0.001.-Age.

The integration of these pathways indicated that perceived social support (*B* = −0.159, *β* = −0.176, *t* = −2.657, *p* < 0.01) significantly and negatively impacted PTSD. While it is noteworthy that hope (*B* = 0.178, *β* = 0.138, *t* = 2.127, *p* < 0.05) positively influenced PTSD. Both pathway descriptions and coefficient values are documented in Table 4; Fig. 2.

**Table 4:** The mediating effect of hope and perceived social support on the relationship between depression and post-traumatic stress disorder in cancer patients.

Regression equations		Integrated fit Index			Regression coefficient significance	
Outcome variables	Predictor variables	*R* ^2^	ΔR²	F	β	t
PTSD	Depression	0.453	0.444	51.918	0.588	10.820***
PTSD	Depression	0.477	0.462	33.866	0.570	9.223***
	Hope				0.138	2.127*
	Perceived social support				−0.176	−2.657**
Hope	Depression	0.194	0.181	15.060	−0.427	−6.467***
Perceived social support	Depression	0.356	0.342	25.831	−0.269	−4.115***
	Hope				0.407	6.228***

Note. * *p* < 0.05, ** *p* < 0.01, *** *p* < 0.001.

### The mediating effects of various pathways through which depression disorder associates PTSD

Table 5 delineates the intervening mechanisms across different routes by which depression is associated with PTSD.

**Table 5:** Results of mediation effect test in the chain mediation model.

	Effect	Boot SE	BootLLCI	BootULCI	z	*p*
Depression-Hope-PTSD	−0.087	0.039	−0.148	0.006	−2.211	0.027
Depression-Perceived Social Support-PTSD	0.069	0.024	0.007	0.101	2.883	0.004
Depression-Hope-Perceived Social Support-PTSD	0.045	0.015	0.004	0.064	2.937	0.003
Direct Effect	0.836	0.091	0.658	1.013	9.19	0.000
Total Effect	0.863	0.080	0.707	1.020	10.79	0.000

Note. SE: Standard Error, CL: Confidence interval; PROCESS(d_demo2, y = “PTSD”, x = “Depression”, meds = c(“perceived social support”, “hope”), covs = c(“age”, “gender”), model = 6, ci= “boot”, nsim = 5000, seed = 1234).

Examining the direct effect between depression and PTSD. The direct pathway from depression to PTSD was crucial, with an effect of 0.836 (*SE* = 0.091, *Z* = 9.19, *95% CI* [0.658 1.013]),which means a 10-point decrease in the SDS corresponds to a 9.1-point reduction on the PCL-C. The analysis demonstrates a small, non-significant indirect effect between depression and PTSD.

Mediation between depression, hope and PTSD. The mediating role of hope failed to reach statistical significance, with an effect of −0.087 (SE = 0.039, *Z* = −2.211, *95% CI* [−0.148, 0.006]).

Mediation between depression, perceived social support and PTSD. The pathway through perceived social support was robust, with an effect of 0.069 (*SE* = 0.024, *Z* = 2.883, *95% CI* [0.007, 0.101]), highlighting perceived social support’s pivotal role in the depression- PTSD nexus.

The chained mediating function of received social assistance and optimism linking depression to PTSD. Within the intricate mediating mechanisms, the interaction of hope and perceived social support demonstrated statistical significance, showing an effect magnitude of 0.45 (*SE* = 0.015, *Z* = 2.934, 95% *CI* = [0.004,0.064]).

The total indirect effect was 0.027 (95% *CI* = [−0.137, 0.171]), and the proportion of the total effect mediated was approximately 3.13%. A large portion (approximately 96.9%) remains unexplained by the studied mediators.

The analytical results illuminate the sophisticated mechanisms linking depression’s impact on PTSD, emphasizing the need to target multiple mediatory mechanisms in interventions aimed at reducing PTSD by addressing depression.

## Discussion

The study’s results suggest that for cancer patients, depression has a significant impact on PTSD, with perceived social support and hope serving as mediating factors. Specifically, perceived social support partially mediates the relationship between depression and PTSD, while no significant mediating effect of hope is observed. Overall, the chain mediation effect, where hope and perceived social support act sequentially, is significant. The findings highlight the significant impact of reducing depression on alleviating PTSD symptoms and suggest that hope and perceived social support play crucial roles in this process.

Our study excluded individuals with psychiatric disorder history to control for confounding variables, allowing better isolation of cancer’s psychological impact on the relationships between depression, hope, social support, and PTSD. However, this exclusion criterion potentially overlooked high-risk groups, limiting generalizability to patients with pre-existing mental health conditions who may experience more severe psychological responses to cancer diagnosis.

The PTSD prevalence among cancer patients was 14.06%, with 27 cases identified out of 192 individuals screened using the PCL-C. This finding is close to the results of a meta-analysis, which reported a prevalence rate of 7.3–13.8% based on self-reported measures, and 12.6% when assessed using standardized clinical structured interviews^41^. However, these results differ from another study, which used the same scale as ours and found that the prevalence of PTSD among adult cancer patients in Oman was 27.4%, significantly higher than in our study^42^. This discrepancy may be attributed to differences in the nationality of the participants and the types of cancer involved. Despite these variations, all studies consistently demonstrate that PTSD occurrence surpasses typical rates within the cancer-affected population^43^.

Our findings revealed that elevated depressive symptoms showed marked correlation with increased PTSD severity, aligning with earlier investigations, which is consistent with previous researches. The present study revealed a significant linear relationship between preoperative and postoperative depression scores and PTSD scores among breast cancer patients^27^. In a previous study, cancer patients were classified into four groups based on their depressive symptomatology. The researchers found that the group with the most severe depression also exhibited more severe PTSD and anxiety disorders compared to other groups^31^. For cancer patients, depressive disorder and PTSD frequently occur as comorbid conditions^23^. A study found that among 287 patients with renal cell carcinoma, 46% exhibited psychiatric symptoms including depression and PTSD, with 15% of patients presenting comorbid depression and PTSD^24^. Our findings corroborated these results, demonstrating a comparable comorbidity rate of 13.5% between depression and PTSD, thus providing additional evidence for the prevalence of psychiatric comorbidity in this clinical population.

Among patients with cancer, our analysis found that perceived social support partially mediated the pathway between depression and PTSD. Individuals experiencing higher levels of depression tended to perceive lower levels of social support. This observation is in accordance with existing literature^44,45^. Notably, Compared to the general population, individuals with cancer demonstrated higher perceptions of social support^46^. There is substantial evidence suggesting that diminished perceived social support represents a crucial risk element in the onset and persistence of PTSD symptomatology^47^. Although cancer patients exhibited enhanced levels of perceived social support compared to healthy controls, individuals with compromised social support perception within this population showed elevated vulnerability to PTSD. In our study, the positive regression coefficient of hope on PTSD contradicts conventional thinking. This may be attributed to the illusory nature of hope. Individuals reporting higher levels of hope may avoid confronting the trauma directly, instead focusing on a seemingly more manageable future. Such a coping mechanism may hinder the genuine processing of trauma, thereby exacerbating PTSD symptoms^48^ and there is no mediating effect of hope between depression and PTSD, which is contrary to our hypothesis. In our study, hope failed to mediate between depression and PTSD, contrary to our hypothesis. While previous research^12^, found hope mediating stress and PTSD in oral cancer patients, this discrepancy likely stems from fundamental differences between perceived stress and depression: stress is situational, whereas depression involves pervasive negative cognitive patterns and emotional numbness that may overwhelm hope’s protective mechanisms. Measurement variations could also contribute to these divergent findings. Interestingly, hope may function as a moderator rather than a mediator in the trauma context. Evidence suggests hope can enhance anxiety’s effect on post-traumatic growth^49^. Thus, hope may influence the strength or direction of depression-PTSD relationships rather than explaining the mechanism. Future research should examine both mediating and moderating models to better understand hope’s role in the depression-PTSD relationship among cancer patients. Moreover, our study found that hope and perceived social support played significant chain mediating roles between depression and PTSD, showing that depression could affect cancer patients’ hope level through perceived social support, and then eventually affected PTSD. Hope could positively predict cancer patients’ perceived social support, which means cancer patients with great hope typically possess stronger psychological resources, enabling them to perceive the help and support from others more positively. The literature documents robust associations between depression and hope levels, suggesting that depressed individuals with diminished hope tend to perceive less social support^50^.We conceptualized depression as a primary distal risk element for PTSD.

The analysis reveals potential mechanisms whereby depression may heighten PTSD because depressive conditions diminish an individual’s sense of hopefulness, thereby reducing their capacity to perceive and accept social support^51^. In the absence of hope, individuals are more inclined to overlook or misinterpret the support offered by others. This mechanism suggests that depression not only directly associates the levels of hopefulness but also indirectly contributes to the worsening of PTSD symptoms by impairing the perception of social support. It deepens insights into PTSD development.

Gender and age, serving as covariates in this study, demonstrated diverse effects across various dimensions of our analysis. Compared with male cancer patients, female cancer patients were more likely to encounter difficulties in perceived social support, which contradicts previous research findings^52^. This inconsistency may be attributed to sample differences, the use of different measurement scales from those employed and variations in cultural backgrounds in this study.This disparity may be attributed to social role conflicts and psychological stigmatization. Traditional female caregiving responsibilities create tension when illness disrupts role fulfillment, while gender-specific body image concerns can heighten stigma. These sociocultural factors compromise women’s perception of available support despite actual resource availability^53^. Meanwhile, consistent with existing literature, female patients were significantly associated with elevated levels of PTSD symptoms^54^. Age also emerged as a significant factor in this study, with older patients exhibiting higher levels of PTSD, which aligns with previous research findings^55^.

## Limitations

Several study limitation require consideration. Firstly, as a cross-sectional study, causal relationships among variables cannot be definitively established. Specifically, PTSD symptoms may not only be a consequence of depression but could also exacerbate depressive symptoms. Additionally, as PTSD, the dependent variable in this study, typically exhibits delayed onset, we could only measure current PTSD levels. Thus, a longitudinal design or alternative statistical approaches, such as structural equation modeling could be proposed in future research to better establish causality.

Secondly, the measurements in this study relied on self-reported scales, which may introduce subjective bias. Particularly for clinical psychiatric conditions such as depression and PTSD, structured clinical interviews could provide more reliable data collection methods.

Thirdly, with cancer patients as our study population, multiple causal confounding factors may be present. Initially, elevated scores on certain items in the PTSD and depression questionnaires might be attributable to the inherent characteristics of specific cancers. For instance, digestive system cancers may lead to difficulties in normal eating, potentially inflating scores for both psychiatric disorders. Furthermore, since cancer and its diagnosis and treatment may precipitate psychiatric disorders, patients’ PTSD symptoms could be caused by cancer rather than depression, and vice versa. Therefore, future research should employ methods to control for cancer-related confounding factors when investigating the relationship between depression and PTSD among cancer patients.

Fourthly, the lack of systematic data collection on cancer types and treatment stages during the preparation phase may have impacted the results and their generalizability, as these factors influence psychological outcomes such as depression, hope, social support, and PTSD. Future studies should address this gap to better understand their effects.

Finally, a notable limitation stems from the relatively small sample size in the present study. The limited number of participants may constrain the statistical power and generalizability of the findings. Future research would benefit from larger-scale investigations to validate these preliminary results and enhance the robustness of the conclusions drawn.

## Clinical implications

These findings provide valuable implications for designing targeted interventions to enhance mental health outcomes in the cancer patient population. For instance, integrated interventions targeting both hope and social support concurrently would be more effective than single-component approaches focusing on hope alone. Clinicians should foster hope within social contexts rather than as an isolated psychological resource, potentially through group-based interventions that leverage social connections. “A study demonstrates that Meaning-Centered Group Psychotherapy (MCGP) significantly improves feelings of hope among advanced cancer patients^56^. Additionally, as depression significantly correlates PTSD development in cancer patients, implementing routine depression screening and early intervention protocols should be prioritized in comprehensive cancer care. Meanwhile, interventions targeting social support enhancement may be more effective than interventions focused solely on hope-building strategies, consistent with the chain mediation model demonstrated in this study.

## Conclusion

The results of this investigation demonstrate that depressive symptoms play a crucial role in diminishing hope levels among cancer patients, subsequently influencing their perception of social support and PTSD symptoms. The mediating effects of hope and perceived social support were observed in the depression-PTSD relationship.

## Electronic supplementary material

Below is the link to the electronic supplementary material.

Supplementary Material 1

## Data Availability

The data that support the findings of this study are not openly available due to reasons of sensitivity and are available from the corresponding author upon reasonable request. Data are located in controlled access data storage at Bengbu Medical University.
