# Supplementary material for: Depression impact on PTSD in Cancer patients through serial mediation of hope and perceived social support
Source: Sci Rep. 2025 Jul 9;15:24727. doi: 10.1038/s41598-025-09908-w (PMC12241477; doi:10.1038/s41598-025-09908-w)
Supplement: Supplementary file 1 — Supplementary Material 1 [file 41598_2025_9908_MOESM1_ESM.docx]

Table S1. Collinearity Diagnosis

| Collinearity Diagnosis | | |
| --- | --- | --- |
| Variable | VIF Value | Tolerance |
| PTSD | 1.707 | 0.586 |
| Perceived Social Support | 1.577 | 0.634 |
| Depression | 1.935 | 0.517 |
| Hope | 1.490 | 0.671 |

Note. Per the reviewer's request, the attached supplementary file provides variance inflation factor (VIF) tables generated from collinearity diagnostics. These tables systematically quantify multicollinearity between predictor variables in our regression models, presenting VIF values for all model specifications referenced in the manuscript.
